# Artificial intelligence (AI) acceptance in primary care during the coronavirus pandemic: What is the role of patients' gender, age and health awareness? A two-phase pilot study

**DOI:** 10.3389/fpubh.2022.931225

**Published:** 2023-01-09

**Authors:** Hila Chalutz Ben-Gal

**Affiliations:** Department of Industrial Engineering and Management, Afeka College of Engineering, Tel Aviv, Israel

**Keywords:** artificial intelligence, digital public health, primary care, coronavirus pandemic, health awareness, pilot study

## Abstract

**Background:**

Artificial intelligence (AI) is steadily entering and transforming the health care and Primary Care (PC) domains. AI-based applications assist physicians in disease detection, medical advice, triage, clinical decision-making, diagnostics and digital public health. Recent literature has explored physicians' perspectives on the potential impact of digital public health on key tasks in PC. However, limited attention has been given to patients' perspectives of AI acceptance in PC, specifically during the coronavirus pandemic. Addressing this research gap, we administered a pilot study to investigate criteria for patients' readiness to use AI-based PC applications by analyzing key factors affecting the adoption of digital public health technology.

**Methods:**

The pilot study utilized a two-phase mixed methods approach. First, we conducted a qualitative study with 18 semi-structured interviews. Second, based on the Technology Readiness and Acceptance Model (TRAM), we conducted an online survey (*n* = 447).

**Results:**

The results indicate that respondents who scored high on innovativeness had a higher level of readiness to use AI-based technology in PC during the coronavirus pandemic. Surprisingly, patients' health awareness and sociodemographic factors, such as age, gender and education, were not significant predictors of AI-based technology acceptance in PC.

**Conclusions:**

This paper makes two major contributions. First, we highlight key social and behavioral determinants of acceptance of AI-enabled health care and PC applications. Second, we propose that to increase the usability of digital public health tools and accelerate patients' AI adoption, in complex digital public health care ecosystems, we call for implementing adaptive, population-specific promotions of AI technologies and applications.

## 1. Introduction

Artificial Intelligence (AI) is a multidisciplinary field of science with the goal of creating intelligent machines (1, 2). AI is steadily entering and transforming various industries. Different industries are in various stages of AI adoption. For example, e-commerce and cybersecurity are considered late adopters, while AI is gradually revolutionizing other industries (3).^1^.

AI has gradually transformed medical practice. Recent progress has been made in the direction of digitized data acquisition, machine learning and computing infrastructure, resulting in AI applications that are steadily entering novel domains that were previously governed solely by human experts. Research has outlined breakthroughs in AI technologies, identified challenges for further progress in health care and medical AI systems (4, 5) and recently analyzed the economic, legal and social implications of AI in health care (3).

Research suggests a transformation in AI in the Primary Care (PC) domain (4). Technological applications based on big data solutions may assist General Practitioners (GP) in disease detection. AI plays a significant role in PC in medical advice, clinical decision-making, diagnostics and digital public health advice (6).

Due to the coronavirus pandemic, health care providers are adjusting health care delivery channels to protect both patients and medical staff from infection through resource allocation directed at new and acute needs. As a result, routine services have stopped or slowed substantially, and strict isolation and separation protocols have been introduced (7).

Prior to the current pandemic, some studies focused on the barriers to using digital public health solutions (8). However, following the coronavirus pandemic, health care providers' treatment of patients with non-urgent and chronic conditions became authoritative. Consequently, video consultation is being introduced, and the use of social media (9) is being discussed for its potential to direct patients to trusted PC resources (7). Nevertheless, some companies (e.g., Babylon Health, Health Tap, Ada, Buoy, Your.MD) have developed AI-powered doctors that provide health advice directly to patients with common symptoms, freeing up PC access for more complex care. By 2025, the market for these services (using the current telemedicine market and retail clinics market as a comparison) is projected to be $27 billion a year (6, 10, 11).

The digital public health care transformation reinforces additional challenges. For example, potential conflicts exist based on patients' sociodemographic backgrounds. Digital tools can provide collective public health benefits; however, they may be intrusive and erode individual freedoms or leave vulnerable populations behind. The coronavirus pandemic has demonstrated the strong potential of various digital solutions (12). The introduction of AI to perform medical tasks remotely contributes immensely to health care and public health domains (6, 13, 14).

In light of recent calls to advance PC with AI and machine learning (15), the goal of this pilot study is to explore patients' readiness to use digital public health solutions in the form of AI-based technology in PC for the purpose of medical advice and diagnostics (16–18). To do that, we focus on some key questions. For example, how likely are patients to use AI-based applications for PC purposes? Which factors delay the adoption of new technological solutions? Which individual perceptions influence patients' potential use of AI? What is the impact of the coronavirus pandemic and forced social distancing on individual attitudes toward AI-based solutions in PC technology adoption?

The study results indicate that patients' privacy concerns, professionalism perceptions, motive perceptions and innovativeness (proneness to technology use score) are all key factors in AI-based technology acceptance in PC during the coronavirus pandemic outbreak. However, we conclude that neither patients' health awareness and empathy needs, nor their sociodemographic factors as described in the TRAM model, such as age, gender and education, are significant predictors of AI-based technology acceptance in PC. Therefore, we suggest exploring the effects of population-specific promoters of individual impediments to accelerate the adoption of AI-based applications in PC to increase their usability in complex digital public health care ecosystems.

## 2. Theoretical background and hypothesis development

### 2.1. Artificial intelligence in primary care

The factors that cause individuals to accept new technologies have been researched over the past few decades. However, AI-based technology adoption, specifically in PC, has not been deeply researched even though, in recent years, the topic of AI in health care has been increasingly investigated. For example, Yu and colleagues (19) presented a review study introducing recent breakthroughs in AI technologies and their biomedical applications with the challenges for medical AI systems in health care. Subsequently, Bini analyzed the impact of AI, machine learning, deep learning, and cognitive computing health care (3). The paper discussed the origin of AI and the progress of machine learning and then discussed how the limitations of machine learning led data scientists to develop artificial networks and algorithms. The study showed how AI can act as a tool to support human cognitive functions for physicians delivering care to patients (3).

AI-based applications have been used in medical imaging of the liver (20), cardiology (21), ophthalmology (22), orthopedics (23) and other medical and PC domains. However, research on AI in PC remains limited. A British study exploring GPs' views on AI and the future of PC (24) explored the potential of AI to disrupt PC and impact key medical tasks (25). This study explored technology and its potential benefits, as well as social and ethical concerns from doctors' perspectives. The study concluded that, from physicians' perspectives, the potential of AI remains limited (24). However, this study explored physicians' perspectives related to AI in PC, leaving patients' perspectives unexplored. Some research related to patients' perspectives was presented by (26). This study utilized online surveys to explore users' attitudes about AI-based medical solutions. The researcher concluded that despite ongoing concerns related to the accuracy of a symptom checker, a large patient-user group perceived the AI-assisted symptom checker to be a useful diagnostic tool. Addressing this research gap reveals that patients' perspectives on the acceptance of AI in PC is a domain to be further explored. Furthermore, no study has analyzed patients' perspectives in the context of the coronavirus pandemic, and such an analysis was therefore the purpose of this study.

AI is utilized to support and improve health services in many high-income countries. There is great hope that AI can also improve health service delivery in resource-poor settings (27). AI-based diagnosis in primary health care may contribute to improving health regulation of the broader health system by technology deployment and scaling up (28). Since gaps in the quality of primary health care still exist, at the primary health care level, specific technology-based clinical care and public health services need to be integrated. With adequate policy regulations, this may contribute to suitable provider payments, health guideline regulations, and health performance assessments, resulting in synergy in health care management (29).

### 2.2. Technology Readiness and Acceptance Model (TRAM)

Our proposed research model examines antecedents extracted from the TRAM model at the individual level through perceived usefulness and perceived ease of use and their effect on readiness to use AI-based mobile applications. The research model aims to explore the influence of privacy, professionalism, empathy, motive, proneness to technology use and health awareness utilizing an individual-level approach.

**Figure 1:**
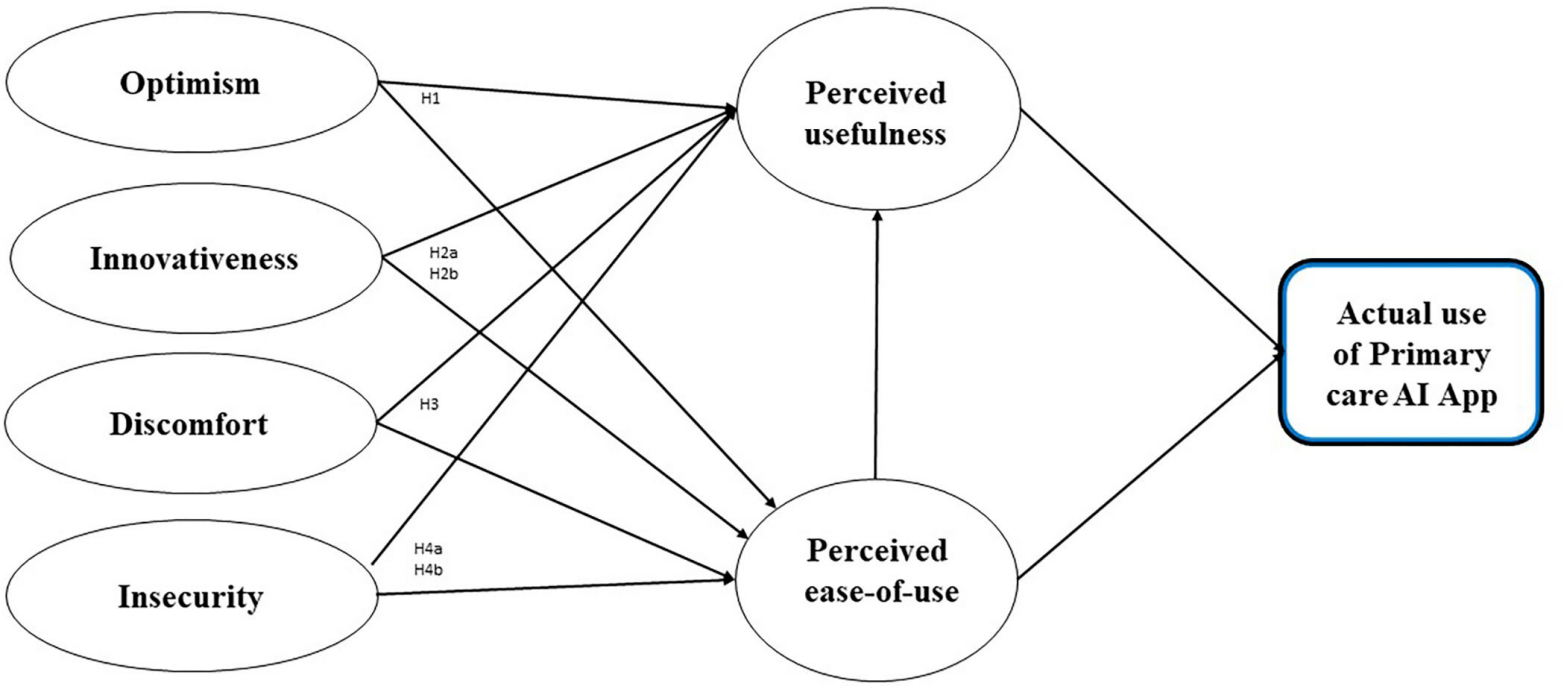
The integrated model (TRAM) with hypothesized relations among study variables.

Figure 1 shows our hypothesized model and the study's theoretical foundation. Our research model emphasizes six core drivers of individual decisions associated with technology readiness and acceptance based on the TRAM model (30, 31). We focus on the six factors depicted in the TRAM model because we believe that they provide a broad perspective and capture the complexity of the new technology acceptance process. Furthermore, exploring all six perspectives enables us to implement a holistic approach to explore the entire AI-based technology acceptance process in PC, considering important elements associated with potential users (6, 31). The proposed research model is based on the integrated TRAM model: readiness to use and adopt AI applications is dependent upon their perception as useful and easy to use. Figure 1 illustrates the TRAM model, which includes four independent variables: optimism, innovativeness, insecurity and discomfort.

### 2.3. Hypotheses development

#### 2.3.1. Hypotheses

In this study, we investigated the potential to use the TRAM model (see Figure 1) to predict patients' readiness to use AI-based applications in PC. We used an adapted version of the TRAM model as developed by Lin et al. (30). Optimistic people generally expect that “good rather than bad things will happen to them” [(32) (p. 219)]. How they approach the world has an impact on their attitudes toward risk perception and acceptance in relation to technology, where optimism relates to a positive view toward technology and trust that it will offer people more efficiency, flexibility and control (33). Building upon this research, we proposed the following hypothesis:

**H1:** Optimism (motive) has a positive influence on readiness to use AI-based applications in PC.

It was stated that “innovativeness” is used to assess the “newness” of an innovation, with innovative products being labeled as having a high degree of newness (34). Parasuraman introduced the technological dimension and referred to “a propensity of being a technology pioneer and influencer” [43, p. 311]. Building upon these insights, we proposed the following hypotheses:

**H2a:** Innovativeness (professionalism) has a positive influence on readiness to use AI-based applications in PC.
**H2b:** Innovativeness (proneness to technology use) has a positive influence on readiness to use AI-based applications in PC.

Discomfort attributes have been defined as “a perceived lack of control regarding technology and the sense of being overwhelmed by it” [43 (p. 311)]. The authors argued that the high-complexity features of technology products have a negative impact on product evaluation because of the user's learning cost (35). Building upon the TRAM model, we propose the following hypothesis:

**H3:** Discomfort (privacy) has a negative influence on readiness to use AI-based applications in PC.

Insecurity “implicates a distrust of technology and the disbelief about its ability to work properly” [43 (p. 311)]. Although the TRAM model suggests that insecurity has a negative impact on the perceived ease of use and perceived usefulness, some recent studies have not been able to find a correlation (28, 36). Building upon the insights of the TRAM model, we proposed the following hypotheses:

**H4a:** Insecurity (empathy) has a negative influence on readiness to use AI-based applications in PC.
**H4b:** Insecurity (health awareness) has a negative influence on readiness to use AI-based applications in PC.

#### 2.3.2. Control variable

We added “referrals to a doctor” as a control variable by asking the respondents for the number of times they have contacted a physician during the past year. According to prior research (37), older adults with certain psychological and health characteristics are more receptive to novel information.

## 3. Method: Data collection and measurement scales

In line with Lancaster et al. (38), the study design and analysis were composed of a two-phase mixed methods pilot study. We intended to measure the effectiveness of utilizing an AI-based application for PC treatment to encourage randomization, which reduces bias and provides a rigorous tool to examine cause-effect relationships (38).

We implemented a two-phase mixed methods research approach (39, 40). First, we conducted a qualitative study (Study 1) that included 18 semi-structured interviews with key job holders in the PC and high-technology industries in Israel, as well as with individual patients. Second, during the coronavirus pandemic, we performed a quantitative study to analyze our research questions that examine the relationship between individual characteristics of patients and their readiness to use AI-based applications in PC (Study 2). By conducting an online survey (*n* = 447), we identified criteria for readiness to use AI-based applications in PC by analyzing the factors that affect the adoption of medical technology based on TRAM. The survey examined six factors that may affect patients' readiness to use AI-based applications in PC: privacy concerns, perception of professionalism, need for empathy, motive perception, proneness to technology use and health awareness (30, 41, 42).

In this study, we determined that the mixed methods technique was the most suitable measurement tool. The mixed methods approach involves data collection and analysis utilizing a mixture of qualitative and quantitative techniques (39, 43). It focuses on collecting, analyzing, and mixing both quantitative and qualitative data in a single study. The central premise of the mixed method procedure is that the combination of both approaches within one study provides a better understanding of the research problems than the use of either approach alone (40). The Tel Aviv University Ethics Institutional Review Board approved the overall study (committee reference number 0001280-1).

### 3.1. Study 1—Qualitative

To validate the research model and gain additional perspective (43), we performed 18 semi-structured interviews with key job holders in the PC and high-technology industries in Israel and with potential patients. The interviews were conducted over a period of ~six months in 2020 (some were conducted face-to-face and some over video calls). The interviewees included eight top executives from the largest Health Management Organization (HMO) in Israel (“Maccabi Health Services”), four top executives from Intel Corporation, a leading high-technology company that leads innovation, digital transformation and AI solutions, and six individual users and patients of the HMO. Each semi-structured interview included nine questions (Appendix A) and lasted ~1 hour; all interviews were recorded, coded and analyzed. The person who interviewed the subjects was also involved in the analysis of the findings.

In line with Lancaster et al. (38), the study design and analysis were carried out based on a two-phase mixed methods pilot study. We intended to measure the effectiveness of utilizing an AI-based application for PC treatment to encourage randomization, which reduces bias and provides a rigorous tool to examine cause-effect relationships (38). Additionally, we undertook precautions to prevent the transfer of bias by interviewing individuals from various organizations. In line with specific recommendations from Lancaster et al. (38), we had a well-defined set of aims and objectives to ensure methodological rigor and scientific validity. For example, the interviewees in Study 1 were not included in Study 2 to ensure the independence of the results of the pilot study.

### 3.2. Study 2—Quantitative

#### 3.2.1. Methodological approach for validation

We used a Confirmatory Factor Analysis (CFA) framework to validate the research variables and the complete structure of the hypothesized model. Specifically, prior to implementing complex indicators, a validation process is necessary to confirm the theoretical constructs, with a complex indicator referring to either a simple or a weighted combination of the original items (44). Seven sets of items were theoretically predefined as research factors, among which three were single-item factors (privacy, professionalism, and motive), one was a two-item factor (empathy), two were four-item factors (proneness to technology use and readiness to use AI-based applications in PC), and one was an eight-item factor (health awareness). For the single-item factors, we built pseudo factors, for which no measurement error was allowed (45, 46). We used a modification process to improve the overall CFA Goodness of Fit (GOF) but minimized this process to remain within the hypothesized theoretical structure (47). Next, we estimated the second-order factors (usefulness and ease of use) within the CFA framework subject to highly correlated factors. The validation process included the exclusion of items that resulted in poor loadings on the theoretical constructs. In addition to construct validity, we examined convergent and discriminant validity. A final hypothesis-testing model was built within the first-order factor structure due to failure to fit the hypothesized second-order latent factors (Figure 3). We applied a structural equation modeling approach to test our hypotheses. A structural equation model is a model of multiple regression equations that allows more than a single outcome variable and indirect effects as part of the model structure (47, 48). All analyses were performed using Mplus version 8.1.

#### 3.2.2. Method: Data collection and measurement scales

To conduct robust and comprehensive research, we focused on quantitative data collection. We utilized technology to launch internet surveys that were emailed to key stakeholders (49). To ensure an appropriate response rate, we used two methods for data collection: web surveys and digital surveys distributed *via* social media. This approach yielded an acceptable and varying response rate (50, 51) of ~40%. Quantitative data were collected in two waves during the coronavirus pandemic from individuals working in the public and private sectors. An online questionnaire was developed in Hebrew and translated into English. The online questionnaire was designed such that data were already coded. Survey respondents were recruited using the snowball method (52). This resulted in 610 responses; after the exclusion of incomplete responses, there were a total of 447 usable questionnaires. In line with Lancaster et al. (38), our sample size was sufficient for a pilot study in Israel to determine the required data for the primary outcome measure (38). Finally, our strategy for handling incomplete responses and efforts to ensure that the responses were missing at random were executed in line with Christensen et al. (51).

The study adopted technology readiness measurement items, including a 4-item instrument evaluating an individual's propensity to adopt and use new technologies in PC. The four dimensions of the TRAM, i.e., optimism, innovativeness, insecurity, and discomfort, consist of six measurement items. A five-point Likert scale ranging from 1 = “Strongly agree” to 5 = “Strongly disagree” was used. Given the potential for finer-grained insights to be acquired using qualitative methods, we incorporated a single open-ended question into the survey. Informed consent was obtained for experimentation. All data collection, validation and analyses were verified independently.

## 4. Results

### 4.1. Descriptive statistics

**Figure 2:**
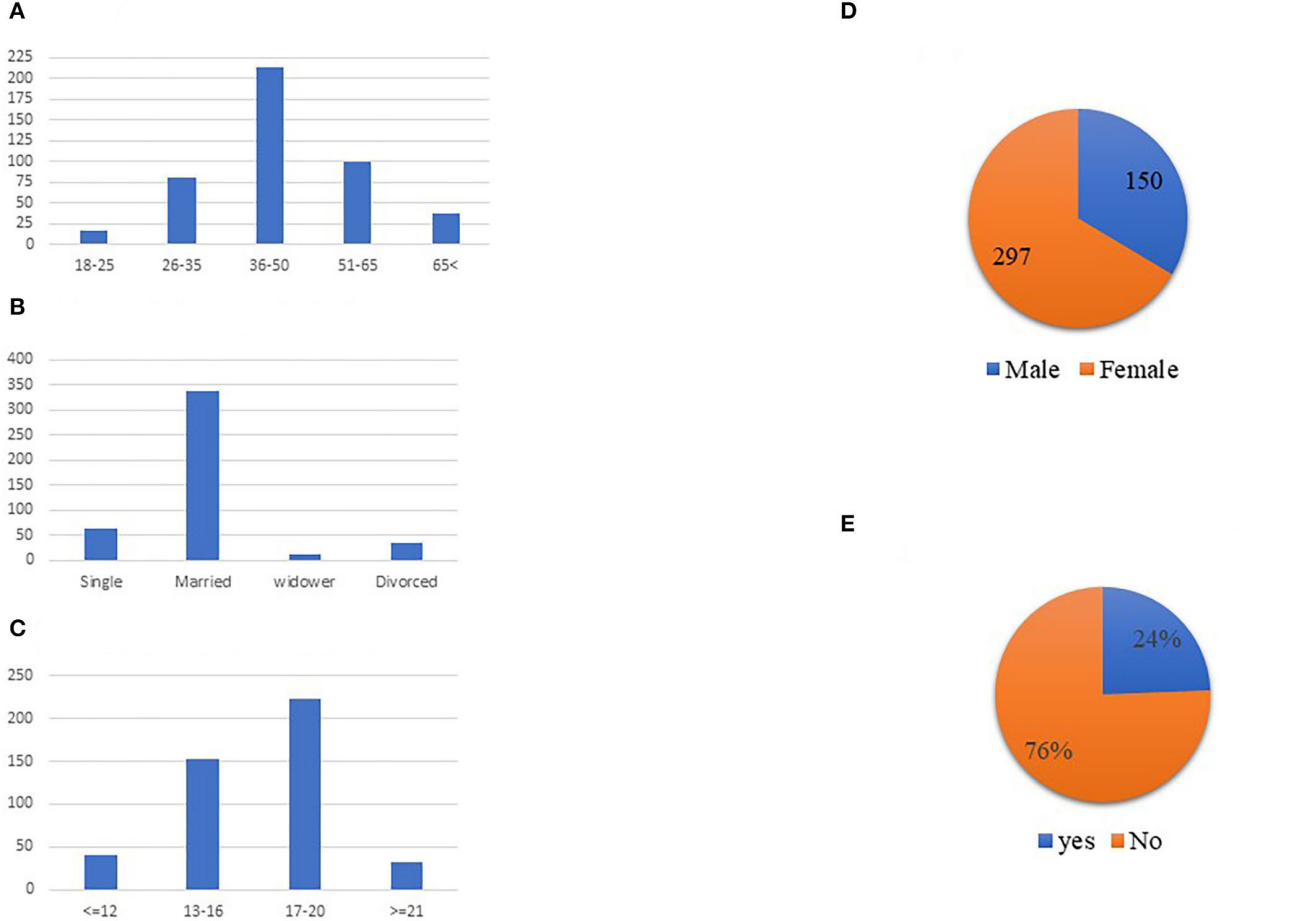
Respondents profile. **(A)** Age distribution. **(B)** Marital status distribution. **(C)** Educational years distribution. **(D)** Gender distribution. **(E)** Do you suffer from chronic illness.

Four hundred and forty-seven respondents completed our questionnaire (66% female and 33% male). The ages of the respondents ranged from eighteen to eighty-five. The average respondent age was 46.09 (SD 0.63), with 4% of individuals aged <25, 18% aged between 26 and 35, 48% aged between 36 and 50, 22% aged between 51 and 65, and 8% aged over 65 years old. Thirty-four percent of respondents had a bachelor's degree, 50% had a master's degree, and 7% had a PhD (see Figure 2).

Sixty-five percent of respondents were employed, 14% were self-employed, 9% were retired, 5% were unemployed, and an additional 7% were unemployed or on leave due to the coronavirus pandemic. The results show that 80% of the respondents in this sample were born in Israel. The vast majority of respondents reported being married (75.3%) and not having any chronic disease (76%). Regarding HMO distribution, half (50%) of respondents obtained their health services from “Maccabi Health Services” and an additional 40% obtained their health services from “Clalit Health Services” – the two largest HMOs in Israel.

### 4.2. Validity and reliability

We tested the construct validity of the TRAM factors within a measurement model. Specifically, a measurement model with six latent constructs and four observed variables was fitted using Mplus version 8.1 (53). We evaluated the model fit utilizing the Robust Root Mean Square Error of Approximation (RMSEA), the Robust Comparative Fit Index (CFI) and the Tucker-Lewis Index (TLI). A CFI larger than 0.95 and an RMSEA value of.05 or lower indicate a good fit. However, small deviations from these standards are acceptable (54). Discriminant and convergent validities were assessed based on correlations across factors (55).

Reliability was measured based on the Cronbach's alpha coefficients of the constructs (2, 56). As a rule of thumb, a Cronbach's alpha coefficient >0.7 is considered acceptable. We concluded that the values indicated acceptable reliability (see Table 1). While performing the CFA, we encountered low GOF, partially due to low item loadings and non-estimated item correlations. We modified the CFA model by excluding items extracted from the health awareness factor (How many times did you feel sick? How many times did you go to the family physician?). This correlation estimation somewhat improved the overall GOF and factor loadings.

**Table 1:** CFA results, factor loadings, and goodness of fit, with item labels.

**Factor**	**Factor and item labels**	**Loading**
F1	Privacy	
	I agree to disclose medical data and answer personal questions related to my health condition in the smart app	1.00
F2	**Professionalism**	
	I believe that a smart app based on my health conditions and on the medical data of many people can provide an accurate medical diagnosis	1.00
F3	**Empathy**	
	When I feel sick, the personal human attitude of the doctor is important to me	0.986
	Since the COVID outbreak, when I feel sick, the personal human attitude of the physician is important to me	0.746
F4	**Motive**	
	I believe the HMO offers an AI-based app for medical diagnostic service to improve the quality of service to its insured	1.00
F5	**Proneness to Technology Use**	
	How often do you use HMO online services for scheduling or locating a service?	0.791
	How often do you use HMO online services for inquiry with your doctor?	0.826
	How often do you use a mobile app for medical diagnosis?	0.301
F6	**Health awareness**	
	Prior to CORONAVIRUS, when I felt sick, I checked my symptoms online and tried to identify the problem myself.	0.545
	Prior to COVID, when I felt sick, I consulted with friends and family	0.498
	During COVID, when I feel sick, I check my symptoms online and try to identify the problem myself	0.470
	During COVID, when I feel sick, I consult with friends and family	0.642
F7	**Readiness to use AI-based applications in PC**	
	To what extent are you willing to use the following services?	
	Doctor consultation by phone	0.779
	Doctor consultation by video call	0.714
	Doctor consultation by chat (e.g., WhatsApp)	0.789
	Doctor consultation via a smart mobile app that knows your medical history	0.772

Fit Indices: χ^2^ = 216.42, *df* = 82, *p* < 0.001; CFI = 0.947, TLI = 0.922; RMSEA = 0.061, 90% CI [0.051.070], SRMR = 0.067; *n* = 447.

Table 1 shows the final CFA results. Several items that were poorly loaded on the latent factor and affected the unacceptable model GOF were dropped during this validation process. The final model had values above the acceptable level for GOF, e.g., CFI = 0.947 and TLI = 0.925. Those factors for which the loading equaled 1.00 were pseudo one-item factors. Although the loading is required to be at least 0.50 in CFA models, we kept the use of mobile apps item in the proneness to technology use factor, as it was essential to the theoretical construct composition. This justification also applied to the health awareness factor. Acceptable construct validity means that the tested model is within a reasonable distance from the empirical data in variance-covariance matrices (53). We also tested the discriminant validity and convergent validity to confirm the unique content of each factor. Our validation was based on the internal consistency - acceptable to high Cronbach's alpha (2, 56) and the model correlations (57), (see Table 2), leading to the conclusion that each factor represented unique and differentiated content. Although the original model suggested mediation between the effects of privacy, professionalism, empathy, motive, and readiness to use AI-based applications in PC through proneness to technology use and health awareness, our empirical analysis did not find such mediation effects. Thus, we continued by modeling the first-order factor effects on the outcome of readiness to use AI-based applications.

**Table 2:** CFA - Correlations between the factors.

**Factor**	**Label**	**F1**	**F2**	**F3**	**F4**	**F5**	**F6**	**F7**
F1	Privacy	-						
F2	Professionalism	0.45^***^ (0.04)	-					
F3	Empathy	−0.13^**^ (0.04)	−0.12^*^ (0.05)	-				
F4	Motive	0.36^***^ (0.04)	0.42^***^ (0.04)	−0.06 (0.05)	-			
F5	Proneness to technology use	0.17^***^ (0.05)	0.07 (0.06)	0.02 (0.06)	0.05 (0.06)	-		
F6	Health awareness	0.16^**^ (0.06)	0.18^*^ (0.08)	−0.05 (0.07)	0.15^*^ (0.07)	0.07 (0.08)	-	
F7	Readiness to use AI-based applications	0.42^***^ (0.05)	0.47^***^ (0.06)	−0.05 (0.05)	0.41^***^ (0.05)	0.25^***^ (0.06)	0.26^***^ (0.07)	-
	Reliability			0.85		0.66	0.76	0.86
	Means	3.21	3.16	3.44	3.66	2.43	2.91	3.56
	SD	1.18	0.99	1.01	0.96	0.86	0.95	0.99

^***^p < 0.001, ^**^p < 0.01, ^*^p < 0.05; standard errors in parentheses; SD, standard deviation; the correlation coefficients were calculated based on the model standard residuals and not as bivariate Pearson's correlation coefficients. The means and SDs represent the calculated mean value indicators and not factor scores.

Table 2 demonstrates the correlations between the factors. As demonstrated in Table 2, privacy concerns, perception of the professional quality of the PC application, motive and technology adaptation were all associated with higher readiness to use AI-based applications in PC. This is demonstrated by the correlation coefficients together with *P*-values, which demonstrate high significance.

### 4.3. Hypothesis testing

#### 4.3.1. Structural model results

**Figure 3:**
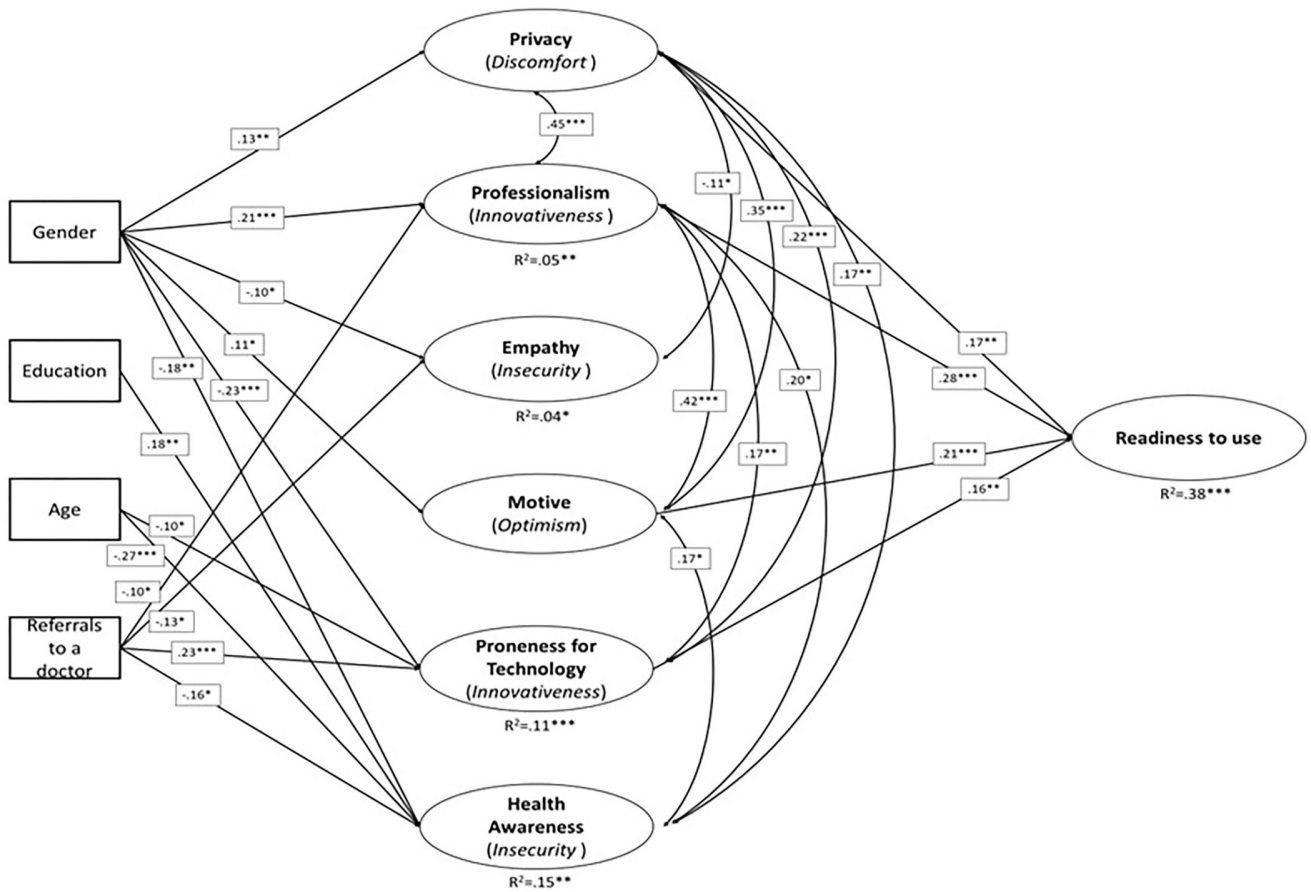
Full illustration of the structural model results.

To test our hypotheses, we built a structural model that included the background variables – gender (men vs. women), education level (years of education), age (five age groups), and number of visits with the family physician (from 1 to 5) (see Table 3). An illustration of the model with significant paths is shown in Figure 3.

**Table 3:** Structural equation model results and standardized regression estimates.

	**F1**	**F2**	**F3**	**F4**	**F5**	**F6**	**F7**
**Variable**	**Privacy**	**Profess**.	**Empathy**	**Motive**	**Proneness to technology use**	**Health aware**	**Readiness to use AI–based applications in PC**
Gender	0.13^**^ (0.06)	0.21^***^ (0.04)	−0.10^*^ (0.05)	0.11^*^ (0.04)	−0.23^***^ (0.05)	−0.18^**^ (0.06)	−0.02 (0.05)
Education	0.06 (0.06)	0.02 (0.05)	−0.02 (0.04)	0.01 (0.05)	−0.02 (0.06)	0.18^**^ (0.06)	0.08 (0.05)
Age	−0.10 (0.05)	0.03 (0.05)	0.07 (0.05)	−0.04 (0.05)	−0.10^*^ (0.05)	−0.27^***^ (0.07)	−0.07 (0.05)
Referral to a doctor	−0.08 (0.05)	−0.10^*^ (0.04)	0.13^**^ (0.04)	−0.07 (0.04)	0.23^***^ (0.04)	−0.16^*^ (0.06)	0.02 (0.05)
Privacy	–	0.45^***^ (0.04)	−0.11^*^ (0.04)	0.35^***^ (0.04)	0.22^***^ (0.05)	0.17^**^ (0.06)	0.17^**^ (0.06)
Professionalism		–	−0.09 (0.05)	0.42^***^ (0.04)	0.17^**^ (0.06)	0.20^*^ (0.08)	0.28^***^ (0.07)
Empathy			–	−0.04 (0.05)	−0.05 (0.06)	−0.03 (0.07)	0.02 (0.04)
Motive				–	0.10 (0.06)	0.17^*^ (0.07)	0.21^***^ (0.06)
Proneness to technology use					–	0.08 (0.08)	0.16^**^ (0.05)
Health awareness						–	0.10 (0.07)
*R2*	0.04 (0.02)	0.05^**^ (0.02)	0.04^*^ (0.02)	0.02 (0.01)	0.11^***^ (0.03)	0.15^**^ (0.05)	0.38^***^ (0.05)

Fit Indices: χ^2^ = 272.19, df = 120, *p* < 0.001; CFI = 0.945, TLI = 0.915; RMSEA = 0.053, 95% CI [0.045, 0.062], SRMR = 0.056. ^***^ *p* < 0.001.

^**^ *p* < 0.01, ^*^ *p* < 0.05; Standard errors in parentheses; Shaded cells for correlations; SD, standard deviation.

We found that gender affected all model factors except the readiness to use AI-based applications in PC. The results indicated that privacy, professionalism and motive were higher among women (β = 0.13, *p* < 0.01; β = 0.21, *p* < 0.001; β = 0.11, *p* < 0.05, respectively), whereas women's results were lower on the empathy, technology, and health awareness factors (β = −0.10, *p* < 0.05; β = −0.23, *p* < 0.001; β = −0.18, *p* < 0.01, respectively). Additionally, a higher education level was associated with higher health awareness, and vice versa (β = 18, *p* < 0.01). However, older age and more frequent visits to the family physician were negatively associated with health awareness (β = −0.27, *p* < 0.001; β = −0.16, *p* < 0.05, respectively). Older respondents were less prone to technology use, as expected (β =- 0.10, *p* < 0.05). However, respondents who made a higher number of visits to the family physician were also more prone to technology use (β = 0.23, *p* < 0.001). A high number of visits to the family physician was negatively associated with professionalism and positively associated with empathy (β = −0.10, *p* < 0.05; β = 0.13, *p* < 0.01, respectively).

Notably, respondents' age and number of visits were somewhat correlated F (4.446) = 2.888, *p* = 0.022; in *post-hoc* comparisons, we found only the 36–50 age group differed from the rest of the age groups, having a smaller number of visits. As demonstrated in Figure 3 and Table 3, the latent factor effects on the outcome factor – readiness to use AI-based application in PC – were positive; that is, higher privacy concerns, perception of the professional quality of the application, motive and technology adaptation were all associated with higher readiness to use (β = 0.17, *p* < 0.01; β = 0.28, *p* < 0.001; β = 0.21, *p* < 0.001; β = 0.16, *p* < 0.01, respectively).

As shown in Figure 3, the overall measurement model showed an adequate fit, with chi-square = 272.19 (df = 120), *p* < 0.001; CFI = 0.925; TLI = 0.915; RMSEA = 0.053; and RMSEA = 0.053.

As demonstrated in Table 3, we found that gender affected all model factors (excluding readiness to use AI-based applications in PC). The results indicated that privacy, professionalism and motive were higher among female respondents. Female respondents scored lower on the empathy, technology, and health awareness factors. As expected, a higher education level was associated with higher health awareness. However, somewhat surprisingly, older age and more frequent visits to the family physician were negatively associated with health awareness. However, respondents who made a higher number of visits to the family physician were also more prone to technology use.

Table 4 provides an overview of the hypothesis test results. H4a and H4b were rejected because the correlation was not statistically significant. Surprisingly, insecurity, which originated from both empathy (H4a) and health awareness (H4b), did not have a negative influence on patients' readiness to use PC applications. This finding might be explained by the fact that health-aware individuals have a greater need for a doctor's human touch than less health-aware patients. Thus, there are other predictors that influence readiness to use AI-based technology in PC (58).

**Table 4:** Hypothesis test results.

**Hypothesis**		**Estimate**	**S.E**.	***Z*-Value**	***P*-value**	**Decision**
H1 motive	→ Readiness to use	0.211	0.048	4.351	^***^	Supported
H2a professionalism	→ Readiness to use	0.270	0.050	5.386	^***^	Supported
H2b proneness to technology use	→ Readiness to use	0.152	0.052	2.918	0.004	Supported
H3 privacy	→ Readiness to use	0.187	0.042	4.451	^***^	Supported
H4a empathy	→ Readiness to use	0.051	0.040	1.279	0.201	Not Supported
H4b health awareness	→ Readiness to use	0.058	0.065	0.880	0.379	Not Supported

*n* = 447; ^***^p < 0.01.

As expected, we found a positive relationship between innovativeness (professionalism and proneness to technology use) and readiness to use AI-based applications (H2a and H2b). This was not surprising, as people who are prone to use technology tend to use AI applications for various usages. Because innovative people are more open to new ideas in general (59), this finding seems plausible. People's innovative attitude has been shown to be an important factor in their adoption of new technologies (60). These people are keen to learn, adopt and utilize new technologies, e.g., AI-based applications in PC, which increases their technology adoption chances (33). We assume that innovative people are more familiar with new technological concepts.

According to the study results, H1 was supported, confirming that optimism (motive) had a positive influence on readiness to use PC applications. The motive represents the individual's belief that the HMO's offers of AI-based applications are indeed intended to improve the quality of service to insured individuals.

Finally, the results supported H3, indicating that discomfort (privacy) was positively correlated with readiness to use AI applications in PC. This finding implies that if individuals are uncomfortable with technology, they will be less likely to use AI-based applications in PC. To conclude, four out of six research hypotheses were supported due to high levels of significance.

## 5. Discussion

AI in health care management is an emerging topic in academia and in practice. However, while physicians' perspectives regarding the utility of AI in PC management have been recently studied (24), patients' perspectives and technology acceptance during the coronavirus pandemic have been underexplored, underpinning the purpose of this study.

Understanding the key social and behavioral determinants of acceptance of AI-enabled public health care and PC applications is of utmost importance. Understanding behavioral models for AI acceptance in public health care is important in accepting alternative approaches to assess patient attitudes and beliefs about AI applications in health care. Exploring patients' perspectives in evaluating and accepting AI-based applications is key to understanding the sources of anxiety and enthusiasm about these emerging technologies. Therefore, the importance of understanding behavioral antecedents to predict how patients are likely to form attitudes and beliefs about medical applications of AI in public health care and PC is important for developing AI tools that match patient needs and anticipate potential patient concerns. This may assist AI developers in aligning patient acceptance to new AI applications, assist in clinical implementation, and direct AI innovation toward those applications for the benefit of patients and the public health care system (61).

Previous research concluded that patients' social context impacts their orientation to utilize AI in health care. It is known that patients' interpretations of their previous experiences with the health care system and non-AI health technology are nested within their broader social context, including social identities and the communities they belong to. These social factors also influence how patients engage with AI in health care. For example, a common social factor is known to be the generational differences in trust in technology (61).

To deepen this investigation, in this article, we explored the potential to use the TRAM model to predict user readiness to use AI-based applications in the PC management domain. Specifically, we examined the relationship between individual characteristics and readiness to use new technology in PC. To our knowledge, this study was the first to apply the TRAM model to investigate patients' perspectives during the coronavirus pandemic.

We detected positive correlations between the respondents' perceptions of HMO motive, perceptions of professionalism, proneness to technology use and privacy and readiness to use AI-based applications in PC. Additionally, our analysis indicated that a portion of the population was ready-to-use AI applications in PC during the coronavirus pandemic. This may be explained by the dependency on technology due to social distancing, fear from contagion and an increased need to examine health status according to symptoms (12).

The AI revolution influences many domains, including health care in general and PC management more specifically (3, 17, 24). Previous research concluded that physicians will continue performing their roles, which remain clinically important despite the increased use of AI, hence contributing to the ongoing care of patients (8, 62). However, there is an emerging need to leverage technology to improve the PC management that patients receive and to assist physicians in providing accurate diagnostics in less time. This research shows that some of the population is ready to use AI applications in PC management, but only if their use will provide professional service, maintain their privacy and not reduce the service level they receive from their HMO today.

Our study results indicated that patients' privacy concerns, perception of professionalism, motive perception and proneness to technology use are all key factors in readiness to use AI-based technology in PC during the coronavirus pandemic. However, we found that health awareness, empathy needs, and patients' sociodemographic factors as described in the TRAM model, such as age, gender and education level, are not significant predictors of readiness to use AI-based technology in PC. Therefore, to increase the usability of digital public health and accelerate patients' AI adoption, exploring the effects of population-specific promoters of and individual impediments to accelerating the adoption of AI-based applications in PC and public health care and increasing usability in complex digital public health care ecosystems is needed (63, 64). Thus, we call for implementing adaptive, population-specific promotions of AI technologies and applications.

AI has the potential to reduce physicians' emotional burden and make them more available for patients, thus enabling a shift from a focus on transactional tasks toward personalized care. Future research can examine the impact of AI technologies in achieving better PC at lower costs and improved wellbeing for physicians and patients alike (6, 25).

Our results may be valuable in a global context. These results may assist policy-makers and possible health institutions, as well as those in the technology industry, in communicating stronger and more effective messages to the public toward a smoother acceptance of new AI-based technologies (65). The impact of having good AI-based diagnostic and other tools in primary health care may benefit some key aspects of public health. Since the public health system is characterized by multiple stakeholders (66), it is specifically important to address key diverse challenges. For example, three stakeholder groups—physicians, hospitals, IT managers and policy-makers—can join forces to maximize the utilization and efficiencies of AI-based technologies for the benefit of public health. Since the perceived challenge by key stakeholders involved in AI technology adoption is not technical (66), it is important to overcome barriers, as these tools may contribute to the public health system as a whole.

Primary health care and AI experts agree that AI has the potential to improve managerial and clinical decisions and processes. Thus, AI adoption in PC may be facilitated by common data standards (1). While the use of AI in medicine should enhance health care delivery, there is a growing need to ensure careful design and evaluation of AI applications. This is specifically important for public health care delivery. Thus, as an integral part of this community, the PC informatics community needs to be proactive by guiding the rigorous development of AI applications such that they will be safe and effective.

AI has the potential to impact the global use of technologies in health care and additional computational AI-based tools in primary health care for the benefit of the entire health care network. Thus, both health care professionals and policy-makers may find the potential for advancing AI-based tools in primary health care (28, 67).

This research is subject to several limitations. First, the five Likert-scale measures we used to measure most of our dependent variables in Study 2 may have captured a limited dimension of these variables (68). Future research might wish to examine additional measures in light of the fact that this study used a variety of research tools following a mixed methods approach, which contributed to its robustness. Second, although the data in this study were in depth and collected *via* two different research tools, they were collected in a single country. However, we tried to overcome this shortcoming in two ways: first, by broadening and enhancing the variety of research tools and therefore performing Study 1 and Study 2; and second, by diversifying our sample in light of the unique coronavirus situation, which influences remote users of AI-based applications (38). However, our diversified sample may indeed be prone to technology. Thus, results regarding this measure should be further investigated. Furthermore, in line with Lancaster et al. (38), and acknowledging that the results from hypothesis testing of a pilot study should be treated as preliminary and interpreted with caution, we call for investigating the study's results on a global scale. For example, it may be that educated individuals may be inclined toward technology usage in general and in health care. Thus, more specific investigations related to how education can be used to predict the usage of AI-based technology acceptance may be insightful.

Finally, the convenience sample survey data are less ideal for external validity and may be subject to common method bias. Since the bias of the sample cannot be measured, inferences based on the convenience sampling were made with regard to the sample itself.

## 6. Conclusions

AI has the potential to impact the global use of technology in health care, and additional computational AI-based tools in primary health care can benefit the entire health care network. Thus, both healthcare professionals, as well as policy-makers, may find the potential in advancing AI-based tools in primary health care (28, 67).

This paper has two major contributions. First, we highlight the key social and behavioral determinants of the acceptance of AI-enabled health care and PC applications. Second, we propose implementing adaptive, population-specific promotions of AI technologies and applications to increase the usability of digital public health and accelerate patients' AI adoption in complex digital public healthcare ecosystems.

## Data availability statement

The original contributions presented in the study are included in the article/supplementary material, further inquiries can be directed to the corresponding author.

## Ethics statement

The studies involving human participants were reviewed and approved by Tel Aviv University. The patients/participants provided their written informed consent to participate in this study.

## Author contributions

HCB: writing—first, second and final drafts, writing—review and editing, validation, writing—final draft, overall supervision, and project supervision.

## Footnotes

^1^List of Abbreviations: AI, Artificial Intelligence; PC, Primary Care; CFA, Confirmatory Factor Analysis; GOF, Goodness of Fit; TRAM, Technology Readiness Acceptance Model; GP, General Practitioner; HMO, Health Management Organization; H, Hypothesis.
